# The impact of individual lifestyle and status on the acquisition of COVID-19: A case—Control study

**DOI:** 10.1371/journal.pone.0241540

**Published:** 2020-11-05

**Authors:** Chang Gao, Zhi Zhao, Fengyuan Li, Jia-lin Liu, Hongyang Xu, Yuanying Zeng, Ling Yang, Jiahao Chen, Xiaoting Lu, Can Wang, Qiang Guo

**Affiliations:** 1 Department of Critical Care Medicine, Suzhou Dushu Lake Hospital (Dushu Lake Hospital Affiliated to Soochow University), Suzhou, Jiangsu, China; 2 Department of Critical Care Medicine, The First Affiliated Hospital of Soochow University, Suzhou, Jiangsu, China; 3 Department of Infectious Disease, The First Affiliated Hospital of Soochow University, Suzhou, Jiangsu, China; 4 Soochow University, Suzhou, Jiangsu, China; 5 Department of Critical Care Medicine, Ruijin Hospital, Shanghai Jiaotong University School of Medicine, Shanghai, China; 6 Department of Critical Care Medicine, WuXi People's Hospital Affiliated to Nanjing Medical University, WuXi, JiangSu, China; 7 Department of Oncology, Suzhou Municipal Hospital, Suzhou, Jingsu, China; 8 Department of Critical Care Medicine, Changshu No.2 People’s Hospital, Suzhou, Jiangsu, China; Qazvin University of Medical Sciences, ISLAMIC REPUBLIC OF IRAN

## Abstract

**Background:**

Coronavirus disease 2019 (COVID-19) has spread to the world. Whether there is an association between lifestyle behaviors and the acquisition of COVID-19 remains unclear.

**Methods:**

In this case-control study, we recruited 105 patients with SARS-CoV-2 infection as a case group from the Wuhan Tongji Hospital (Wuhan, China). For each case two control subjects were recruited. Participants were randomly selected from communities in Wuhan and matched for sex, age (± 2yrs), and pre-existing comorbidities (hypertension and diabetes).

**Results:**

A total of 105 patients diagnosed with COVID-19 and 210 controls were included. Compared with control group, the case group had higher proportions of lack of sleep (30.5% vs. 14.8%, P = 0.001) and increased physical activities (56.2% vs. 32.9%, P < 0.001). And patients in the case group were more likely to have alopecia (28.6% vs. 10.0%, P < 0.001) than people from the control group. Overall, we found that lack of sleep [adjusted odds ratio (OR) 1.56, 95% confidence interval (CI) 1.03–2.39)], physical activities (≥ 5 times a week) (adjusted OR 2.05, 95%CI 1.39–3.02) and alopecia (adjusted OR 1.73, 95%CI 1.13–2.66) were independent risk factors for COVID-19 infection. Conversely, low-dose alcohol intake (<100g alcohol per week), hand hygiene, and fruits intake (daily) were significantly associated with a decrease in morbidity.

**Conclusions:**

Individual lifestyle behaviors and health status can affect the occurrence of COVID-19.

## Introduction

According to the coronavirus disease 2019 (COVID-19) situation dashboard of World Health Organization (WHO), as of May 10, 2020, more than 4,000,000 COVID-19 cases have been confirmed worldwide, with nearly 300,000 deaths. The WHO states that the outbreak of COVID-19 constitutes a public health emergency of international concern [1].

In view of the rapid outbreak of COVID 19, it is necessary to find factors associated to occurrence of the disease in addition to the necessary infection control measures. However, most studies focus on the pathogenesis [2], treatment [3, 4] and symptom relief of COVID-19. We still know little about the influence of lifestyle and health status in COVID-19, which may provide a great value for the uninfected population.

Previous studies reported that, individual lifestyle and health status correlated with other respiratory infection [5–8]. Keeping mouth clean can reduce the risk of pneumonia [5, 7]. People who drink tea regularly have some resistance to the flu virus [6]. Here, we focused on whether lifestyle and health status could impact the occurrence of COVID-19.

## Methods

### Participants

**Fig 1:**
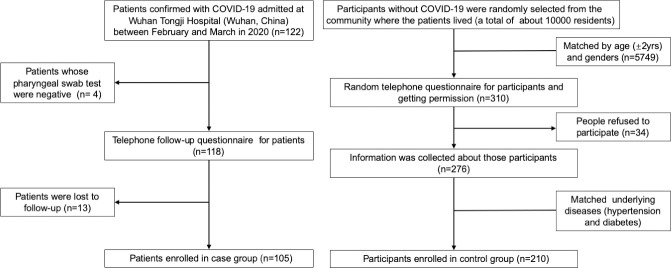
Flow diagram of patients with confirmed COVID-19 and matched participants included in this study.

This Case-Control study was conducted in Wuhan Tongji Hospital (Wuhan, China). We recruited a total of 105 patients aged over 18 years with SARS-CoV-2 infections between February 10, 2020 and March 1, 2020 as a case group. All COVID-19 patients were diagnosed according to World Health Organization interim guidance and the Chinese management guideline for COVID-19 (version 7.0). For each case two control subjects were recruited. Participants in the control group were randomly selected from communities where the patients lived (a total of about 10000 residents) in Wuhan and matched for sex, age (± 2yrs), and underlying diseases (hypertension and diabetes) (Fig 1). All participants in the control group were undiagnosed for COVID-19.

Due to the emergency of the pandemic and the closed management of communities, the written consents were exempted. All participants agreed verbally. The verbal consent was done during the phone call. This consent procedure was approved by the institutional review boards (IRB) at Wuhan Tongji Hospital and The First Affiliated Hospital of Soochow University (2020–054). All data were collected by telephone questionnaires.

### Data collection

The collection of data was divided into two parts. The first part was the basic information of the all participants, including age, sex, and pre-existing comorbidities including hypertension and diabetes. In the case group, 105 participants were obtained by referring to their electronic medical records. In the control group, 210 participants were obtained by questionnaire. We invited local community members to participate in the questionnaire communication process, we conducted standardized training for all staff in advance.

The second part was about participants' lifestyle and health status, which were collected by filling in questionnaires. Lifestyles and health status included smoking status, secondhand smoking exposure (duration per day), alcohol intake (types and doses per week), fruits intake (types and frequency), regular diets, time of falling asleep and getting up in a week, lack of sleep referred to sleep duration <7h per night. (sleep duration = (5 × weekday sleep duration) + (2 × weekend sleep duration)/7) [9–12], lunch break (frequency per week), physical activities (frequency per week in latest 2 months), hand hygiene, living alone, spicy food intake (frequency per week), playing Mahjong (frequency per week), cooking by oneself (frequency per week), alopecia [13], constipation [14], common cold [15] (frequency per year).

### Statistical analysis

If continuous data showed a skewed distribution, they were presented as median [interquartile range (IQR)]. Frequency data were expressed as proportions. Comparisons of continuous variables were made with Student’s t test or the Mann-Whitney U test when appropriate, while differences in categorical variables were assessed using the χ^2^ test or Fisher’s exact test, as appropriate.

Multivariate conditional logistic regression models were used to determine the independent risk factors for COVID-19 occurrence. Variables with *P* < 0.1 in univariate logistic regression (Table 2) were included in the multivariate model. Probabilities of entering and removing variables in a stepwise manner in the multivariate model were 0.05 and 0.10, respectively. Data were analyzed using SPSS 25.0 (IBM, Chicago, IL, USA). Statistical charts were performed using GraphPad Prism 7 (GraphPad Software, San Diego, CA, USA). A two-tailed P value < 0.05 was considered statistically significant.

## Results

A total of 105 patients diagnosed with COVID-19 and 210 controls matched for age, sex and pre-existing comorbidities (hypertension, diabetes) were included in this study (Fig 1). The median age was 55.0 years old (IQR, 45.5–66.5) in case group, and 54.0 years old (IQR, 45.0–68.0) in control group (*P* = 0.889). 45.7% of the people were male, 19% and 2.9% with hypertension and diabetes, respectively (Table 1).

**Table 1:** Comparison of baseline, living habits and health status between case group and control group.

Factor	Case (n = 105)	Control (n = 210)	*P*
Age, median (IQR), yr	55.0 (45.5–66.5)	54.0 (45.0–68.0)	0.889
Male, n (%)	48 (45.7)	96 (45.7)	
Hypertension ^*a*^, n (%)	20 (19.0)	40 (19.0)	
Diabetes ^*a*^, n (%)	3 (2.9)	6 (2.9)	
Current smokers, n (%)	23 (21.9)	52 (24.8)	0.575
Secondhand smoking exposure ^*b*^, n (%)	22 (21.0)	58 (27.6)	0.200
Low-dose alcohol intake ^*c*^, n (%)	11 (10.5)	45 (21.4)	0.017
Fruits intake daily, n (%)	51 (48.6)	172 (81.9)	<0.001
Regular diets, n (%)	73 (69.5)	181 (86.2)	<0.001
Lack of sleep ^*d*^, n (%)	32 (30.5)	31 (14.8)	0.001
Lunch break daily, n (%)	59 (56.2)	147 (70.0)	0.015
Physical activities ^*e*^, n (%)	59 (56.2)	69 (32.9)	<0.001
Hand hygiene, n (%)	49 (46.7)	146 (69.5)	<0.001
Living alone, n (%)	10 (9.5)	22 (10.5)	0.792
Spicy food intake ^*f*^, n (%)	39 (37.1)	58 (27.6)	0.084
Playing Mahjong ^*g*^, n (%)	32 (30.5)	64 (30.5)	1.000
Cooking by oneself^*e*^, n (%)	62 (59.0)	143 (68.1)	0.112
Alopecia, n (%)	30 (28.6)	21 (10.0)	<0.001
Constipation, n (%)	21 (20.0)	40 (19.0)	0.840
Common cold ≥ 3 times a year, n (%)	12 (11.4)	23 (11.0)	0.899

^*a*^ Pre-existing condition.

^*b*^ Duration of secondhand smoking exposure ≥ 1h per day.

^*c*^ No more than 100g alcohol intake per week.

^*d*^ Lack of sleep referred to sleep duration <7h per night.

^*e*^ Equal to or more than 5 times a week.

^*f*^ Equal to or more than 3 times a week.

^*g*^ Equal to or more than once a week.

Abbreviation: IQR, interquartile range.

In terms of individual lifestyle, the case group had significantly higher proportions of lack of sleep (30.5% vs. 14.8%, *P* = 0.001), and significantly more frequent physical activities (56.2% vs. 32.9%, *P* < 0.001), but lower proportions of low-dose alcohol intake (10.5% vs. 21.4%, *P* = 0.017), fruits intake daily (48.6% vs. 81.9%, *P <* 0.001), regular diets (69.5% vs. 86.2%, *P <* 0.001), good practice of hand hygiene (46.7% vs. 69.5%, *P* < 0.001), and lunch break (56.2% vs. 70.0%, *P* = 0.015). In terms of health status, patients in the case group were more likely to have alopecia (28.6% vs. 10.0%, *P <* 0.001) than people from the control group. There was no statistical difference in the second-hand smoking exposure, spicy food intake, living alone, playing Mahjong, constipation, cooking by oneself, or catching common cold ≥ three times one year between the two groups (Table 1).

In the univariate conditional logistic regression model, low-dose alcohol intake, fruits intake daily, regular diets, lack of sleep, physical activities, lunch break, hand hygiene and alopecia were significant associated with illness occurrence (Table 2). Factors with *P* < 0.1 in univariate logistic regression were included in the multivariate model. Probabilities of entering and removing variables in a stepwise manner in the multivariate model were 0.05 and 0.10, respectively.

**Table 2:** Univariate conditional logistic regression analysis of factors associated with COVID-19 occurrence.

Factor	Unadjusted OR (95% CI)	*P*
Current smokers	0.83 (0.46–1.52)	0.545
Secondhand smoking exposure ≥ 1h per day	0.66 (0.3–1.20)	0.168
Low-dose alcohol intake ^*a*^	0.37 (0.17–0.81)	0.013
Fruits intake daily	0.20 (0.12–0.36)	<0.001
Regular diets	0.32 (0.17–0.60)	<0.001
Lack of sleep ^*b*^	1.75 (1.16–2.66)	0.008
Lunch break daily	0.55 (0.33–0.90)	0.017
Physical activities ≥ 5 times a week	2.45 (1.53–3.93)	<0.001
Hand hygiene	0.38 (0.23–0.63)	<0.001
Living alone	0.89 (0.39–2.04)	0.782
Spicy food intake ≥ 3 times a week	1.62 (0.95–2.76)	0.074
Playing Mahjong ≥ once a week	1.00 (0.60–1.66)	1.000
Cooking by oneself ≥ 5 times a week	0.65 (0.39–1.09)	0.654
Alopecia	3.63 (1.90–6.92)	<0.001
Constipation	1.07 (0.57–2.02)	0.829
Common cold ≥ 3 times a year	1.06 (0.46–2.47)	0.885

^*a*^ No more than 100g alcohol intake per week.

^*b*^ Lack of sleep referred to sleep duration <7h per night.

Abbreviation: OR, odds ratio.

**Fig 2:**
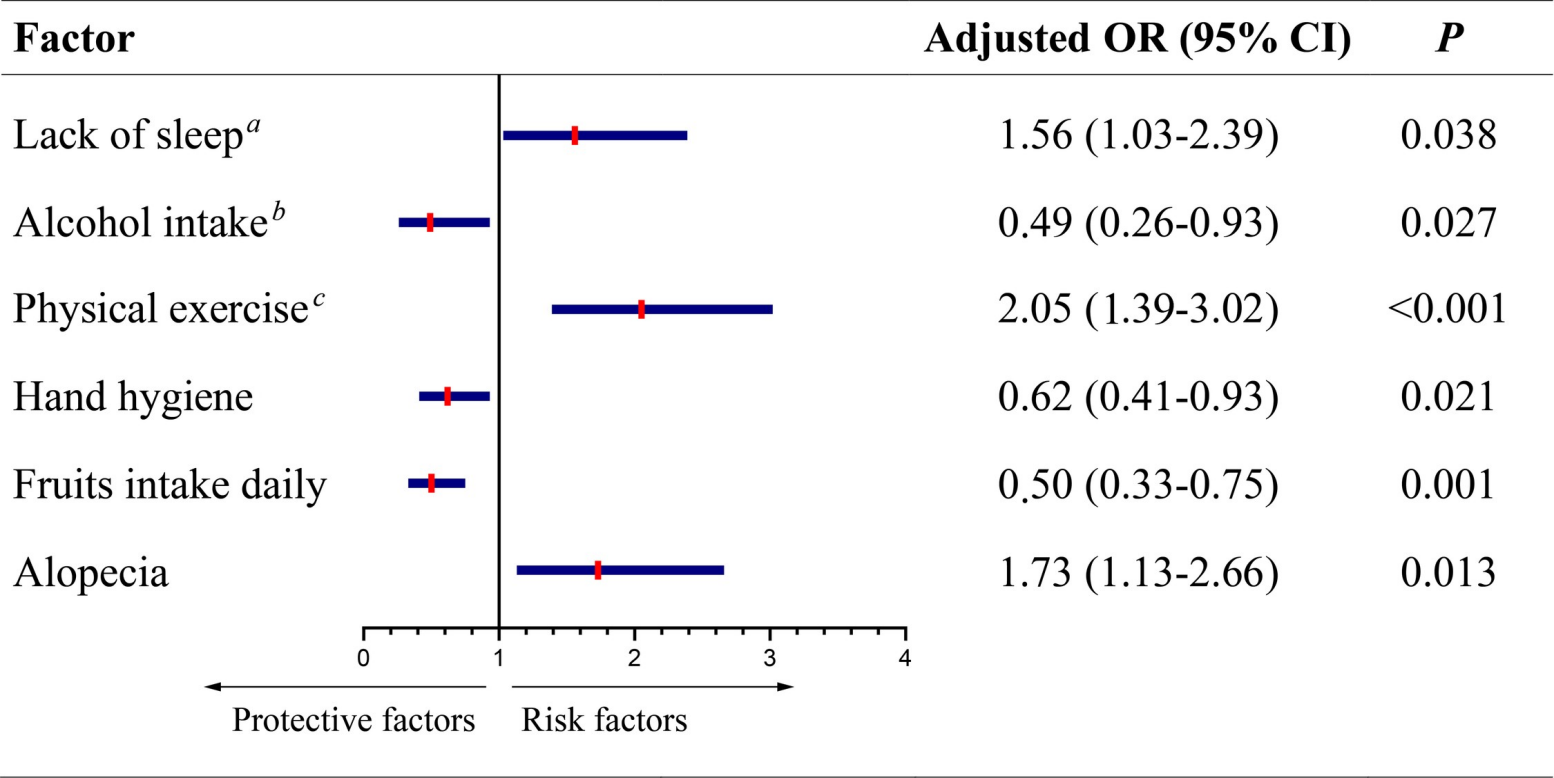
Multivariate Cox regression analysis of individual lifestyles or health status and the COVID-19 infection. ^*a*^ Lack of sleep referred to sleep duration <7h per night. ^*b*^ No more than 100g alcohol intake per week. ^*c*^ Equal to or more than 5 times a week.

In the multivariate analysis, 3 variables were independently associated with the COVID-19 infection: lack of sleep [adjusted odds ratio (OR) 1.56, 95% confidence interval (CI) 1.03–2.39)], physical activities (≥ 5 times a week) (adjusted OR 2.05, 95% CI 1.39–3.02) and alopecia (adjusted OR 1.73, 95% CI 1.13–2.66). In contrast, low-dose alcohol intake (<100g alcohol per week), hand hygiene, and fruits intake (daily) significantly associated with a decrease in morbidity (adjusted OR 0.49, 95% CI 0.26–0.93), (adjusted OR 0.62, 95%CI 0.41–0.93), and (adjusted OR 0.50, 95% CI 0.33–0.75), respectively (Fig 2).

## Discussion

To the best of our knowledge, this study is the first case-control study to focus on the association between individual lifestyle and health status and COVID-19 occurrence. Now that the number of people infected with COVID-19 is on the rise in many countries around the world. WHO declared a public health emergency of international concern over the global outbreak of novel coronavirus [16]. The Managing Director of the International Monetary Fund (IMF) mentioned: We know this is not just a public health crisis. We are aware of the serious social and economic consequences of the pandemic. In addition to isolation and aggressive treatment of confirmed patients, it is also critical to identify factors that affect the adult infection and to implement active and effective interventions.

Sleep is a physiological process that regulates function and has potential effects on human immunity [17, 18]. Lack of sleep usually means disrupted sleep and shorter hours, which can reduce the body's immunity and increase the risk of infection. A previous study showed that, during partial sleep deprivation, human body will lead to a series of physiological changes in the body, including impaired mitogenic proliferation of lymphocytes and decreased HLA-DR expression, thus increasing the susceptibility to viruses [19]. This might explain the finding that lack of sleep was one of the independent risk factors for covid-19 occurrence in our study. Lack of sleep has a negative effect on the overall functioning of human bodies [20]. High negative emotions seem related to a bad night of sleep, and lack of sleep can worsen emotional disorders [21, 22].

Normally, we think physical exercise is good for health. However, we found that an increased risk of SARS-CoV-2 infection in adults was related to physical activity, which was a very interesting result. Physical activity has important physiological effects on the body's immune system [23, 24], but its impact on the risk of respiratory infections is controversial. In addition, people often chose gyms for physical exercise in winter, where the poor ventilation of the environment may increase the exposure of asymptomatic COVID-19 patients and virus outside.

One of the most common forms of hair loss in men is androgenic alopecia [25]. Data mining showed that males were more likely to be infected with COVID-19 than females, and adults were far more likely to be infected than children under 14 years old [26]. A recent study reported that androgen-AR signaling could regulate the ACE2/TMPRSS2 expression and was involved in the macrophage polarization, in addition, the blockage of AR signaling with AR antagonist GT0918 reduced ACE-2 and TMPRSS2 expression and inhibited the expression of macrophage activation makers and TNF-α [27]. Therefore, it can be inferred that men with androgenic hair loss are more likely to be infected with COVID-19 [28], which is also consistent with the results of our finding.

We found that good hand hygiene habits were associated with a decrease in the incidence of infection. SARS-CoV-2 can survive outside the body for a long time. It can survive on plastic for three days, can survive for several days in stainless steel. Some scholars suggest that special attention should be paid to washing hands before sneezing, coughing, exposure in public toilets or eating [29], because proper and effective hand hygiene can reduce an individual exposure to viruses [30].

The WHO has issued a statement saying: "The low-dose of alcohol intake is good for health" has no scientific basis. A systematic analysis for the Global Burden of Disease Study 2016 showed that, alcohol use was a leading risk factor for global disease burden and causes substantial health loss [31]. Whether low-dose alcohol intake can bring benefits to the prevention of infection is unclear. However, we do not recommend low-dose drinking to prevent COVID-19 due to its health risks.

Fruits (such as apples, oranges) intake daily was considered as one of the protective factors for COVID-19 infection in this study. It is well accepted that fruits are rich in vitamins and trace elements. Vitamins can induce the production of antimicrobial peptides, which promote antibacterial activity against a range of microorganisms, thus enhancing immunity [32, 33]. It has been reported that in COVID-19 patients, the immune system produces pro-inflammatory and anti-inflammatory cytokines in response to viral infections [34], and vitamin can reduce the production of pro-inflammatory cytokines while increasing the expression of anti-inflammatory cytokines [35]. Previous studies have shown an important link between vitamin C and infection [36, 37]. Vitamin C has been shown to enhance differentiation and proliferation of B- and T-cells, supplementation with vitamin C appears to be able to both prevent and treat respiratory and systemic infections [38]. Additionally, people with vitamin A deficiency are more likely to have an increased risk of having an impaired immune response to respiratory and influenza viruses [39, 40]. These are consistent with the results of our study, and can partially explain its mechanism.

This study is limited by its retrospective nature, which cannot represent all the COVID-19 patients, but we did find that individual lifestyles and health status could impact the morbidity. Secondly, due to the epidemic management, the questionnaire was completed by telephone, so we could not get more details of the living habits and health status included in this study.

## Conclusion

Individual lifestyles and health status can affect the occurrence of COVID-19, which has a certain degree of significance for the prevention of epidemic. Larger studies are needed to confirm these conclusions.

## Supporting information

S1 Questionnaire (DOCX)
