# Supplementary material for: The impact of individual lifestyle and status on the acquisition of COVID-19: A case—Control study
Source: PLoS One. 2020 Nov 5;15(11):e0241540. doi: 10.1371/journal.pone.0241540 (PMC7643946; doi:10.1371/journal.pone.0241540)
Supplement: S1 Questionnaire — (DOCX) [file pone.0241540.s001.docx]

**新冠患者个人生活方式问卷调查表**

**填表单位：**

**所 在 地： （省、自治区、直辖市）**

**填表日期： 年 月 日**

**填表说明**

**一、本调查表由医疗机构填写。因疫情原因无法由医疗机构人员调查填写，则需要经由医疗机构培训的相关社区人员调查填写。**

**二、调查表内容的填写应真实、准确、完整，字迹清晰。**

新冠患者个人生活方式问卷调查表

调查日期： 问卷序列号：

| 所属医疗机构名称 |  | | | |
| --- | --- | --- | --- | --- |
| 填表人员信息 | 姓名 |  | 工作单位 |  |
|  | 职务 |  | 职称 |  |
|  | 电话 |  | E-mail |  |
| 受试者招募方式（自行招募/CRO/其他） |  | | | |
| 受试者信息 | 姓名 |  | 性别 |  |
|  | 年龄 |  | 工作/学习单位 |  |
|  | 居住地址 |  | 职业 |  |
|  | 联系方式 |  | 当地/外来居民人员 |  |
|  | 备注 |  | | |

| 调查问题  （括号内容及不适用内容可回答在备注中） | 回答结果 | | | 备注 |
| --- | --- | --- | --- | --- |
| 1、 请问您是否有高血压的病史？ | 是 | 否 | 不适用 |  |
| 2、 请问您是否有糖尿病的病史？ | 是 | 否 | 不适用 |  |
| 3、 请问您是否吸烟？ | 是 | 否 | 不适用 |  |
| 4、是否每日暴露二手烟 | 是 | 否 | 不适用 |  |
| 4．1、 请问您每天暴露在二手烟的时间是否等于或超过1个小时？ | 是 | 否 | 不适用 |  |
| 5、 请问您每周的是否饮酒？ | 是 | 否 | 不适用 |  |
| 5.1、饮酒种类 | 啤酒 | 白酒 | 红酒 | 其他请说明： |
| 5.2、饮酒频率（次/周） | ________________ 次/周 | | |  |
| 5.3、每次饮酒量（ml） | 啤酒  ml | 白酒  ml | 红酒  ml | 其他请说明： |
| 6、 请问您每天是否会吃水果？（种类和频率） | 是 | 否 | 不适用 |  |
| 6.1、水果种类（填写） | 1. | 2. | 3. |  |
| 6.2、水果量（个数） | 1.___个 | 2.__个 | 3.___个 |  |
| 7、 请问您每天是否会按时午休？ | 是 | 否 | 不适用 |  |
| 8、 请问您三餐饮食时间是否规律？ | 是 | 否 | 不适用 |  |
| 9、请问您每天晚上几点睡觉？ | ___________________点 | | |  |
| 10、请问您每天早上几点起床？ | ___________________点 | | |  |
| 11、请问您每周都进行体育锻炼？ | 是 | 否 | 不适用 |  |
| 11.1、请问您每周都进行体育锻炼的次数是否等于或大于五次？（运动地点） | 是 | 否 | 不适用 |  |
| 11.2运动场所 | 户外 | 健身房 |  | 其他请说明： |
| 12、请问您平时是否注重手卫生？（外出回家、饭前、便后洗手） | 是 | 否 | 不适用 |  |
| 13、请问您是否长期独自生活吗？（6个月及以上） | 是 | 否 | 不适用 |  |
| 14、请问您吃辛辣的食物吗？ | 是 | 否 | 不适用 |  |
| 14.1、请问您每周吃辛辣的食物频率 | _________________次/周 | | |  |
| 15、请问您一周打麻将的次数是否等于或大于一次？ | 是 | 否 | 不适用 |  |
| 16、请问您每周自己做饭的天数是否等于或大于五次？ | 是 | 否 | 不适用 |  |
| 17、请问您是否有脱发^*^的症状？（由拉发实验判断，详见后说明） | 是 | 否 | 不适用 |  |
| 18、请问您在日常生活中是否经常容易生气发脾气？ | 是 | 否 | 不适用 |  |
| 19、请问您是否有便秘的症状？（便秘指便秘是指大便次数减少或排便不畅、费力、困难、粪便干结） | 是 | 否 | 不适用 |  |
| 20、请问您是否经常感冒？（频率）（感冒指上呼吸道疾病，典型症状包括鼻塞和流涕，打喷嚏，喉咙痛和咳嗽） | 是 | 否 | 不适用 |  |
| 20.1、每年大概感冒几次？ | ________________次/年 | | |  |

^*^拉发实验：用拇指、食指和中指，从头发根部轻轻捏住约40-60根头发，顺着头发方向捋。如果只有1-2根头发脱落，表示正常。如果每次拉发脱发超过5根，则判断实验阳性，怀疑存在异常脱发。

Individual Lifestyle and Status questionnaire on of COVID-19 patients

| Question | Answers | | | Notes |
| --- | --- | --- | --- | --- |
| 1. Do you have a history of hypertension? | Yes | No | Inapplicable |  |
| 2. Do you have a history of diabetes? | Yes | No | Inapplicable |  |
| 3.Are you a current smoker? | Yes | No | Inapplicable |  |
| 4、Are you exposure to second-hand smoke daily? | Yes | No | Inapplicable |  |
| 4.1 Is your daily exposure to second-hand smoke equal to or more than 1h? | Yes | No | Inapplicable |  |
| 5. Do you drink weekly? | Yes | No | Inapplicable |  |
| 5.1 Types | Beer | White wine | Red wine |  |
| 5.2 Frequency（times/week） | ________________ times/week | | |  |
| 5.3 How much do you drink?（ml） | Beer  ml | White wine  ml | Red wine  ml |  |
| 6. Do you eat fruit daily? (types and frequency） | Yes | No | Inapplicable |  |
| 6.1 Types | 1. | 2. | 3. |  |
| 6.2 How much do you eat? (numbers of each types) | 1.___ | 2.__ | 3.___ |  |
| 7. Do you take your lunch break on time every day? | Yes | No | Inapplicable |  |
| 8. Do you eat regularly？ | Yes | No | Inapplicable |  |
| 9.What time do you go to bed every night? | ___________________o’clock | | |  |
| 10.What time do you get up in the morning? | ___________________ o’clock | | |  |
| 11. Do you do physical exercise regular? | Yes | No | Inapplicable |  |
| 11.1Do you do physical exercise equal to or more than five times a week? | Yes | No | Inapplicable |  |
| 11.2 Place | Outdoor | Gym |  |  |
| 12. Do you have good hand hygiene?（Wash your hands when going home, before eating, after using the toilet） | Yes | No | Inapplicable |  |
| 13. Do you live alone for over 6 months? | Yes | No | Inapplicable |  |
| 14.Do you eat spicy food? | Yes | No | Inapplicable |  |
| 14.1、How many times do you eat spicy food a week? | _________________times/week | | |  |
| 15. Do you play mahjong equal to or more than one times a week? | Yes | No | Inapplicable |  |
| 16. Do you cook by yourself equal to or more than one times a week? | Yes | No | Inapplicable |  |
| 17. Do you have any symptoms of hair loss*?(The pull test) | Yes | No | Inapplicable |  |
| 18. Do you get angry easily in your life? | Yes | No | Inapplicable |  |
| 19. Do you have symptoms of constipation recently?(Infrequent passage of stools or  difficulty with evacuation of stools.) | Yes | No | Inapplicable |  |
| 20.Do you get the common cold ofen?（The common cold is a conventional term for a mild upper respiratory illness, the hallmark symptoms of which are nasal stuffiness and discharge, sneezing, sore throat, and cough ） | Yes | No | Inapplicable |  |
| 20.1、How many times do you get common cold per year？ | ________________times/year | | |  |

*The pull test may be used to diagnose hair loss conditions. The examiner grasps approximately 40 to 60 hairs at their base using the thumb, index, and middle fingers and applies gentle traction away from the scalp. A positive result is when more than five are pulled from the scalp; this implies active hair shedding and suggests a diagnosis of telogen effluvium, anagen effluvium, or alopecia areata.
